# Crystal structure of (*E*)-*N* ^1^-[(anthracen-9-yl)methyl­idene]-*N* ^4^-phenyl­benzene-1,4-di­amine

**DOI:** 10.1107/S2056989016020612

**Published:** 2017-01-10

**Authors:** Md. Serajul Haque Faizi, Ashanul Haque, Musheer Ahmad, Irina A. Golenya

**Affiliations:** a Department of Chemistry, College of Science, Sultan Qaboos University, PO Box 36 Al-Khod 123, Muscat, Sultanate of Oman; b Department of Applied Chemistry, Aligarh Muslim University 202 002 UP , India; c National Taras Shevchenko University, Department of Chemistry, Volodymyrska str. 64, 01601 Kyiv, Ukraine

**Keywords:** crystal structure, hydrogen bonding, Schiff base, C—H⋯π inter­actions, 9-anthraldehyde, *N*-phenyl-*p*-phenyl­enedi­amine, AMPD, anthracene

## Abstract

The title compound, C_27_H_20_N_2_, a Schiff base synthesized *via* a condensation reaction between anthracene-9-carbaldehyde and *N*-phenyl-*p*-phenyl­enedi­amine, crystallizes with three independent mol­ecules in the asymmetric unit. The three mol­ecules have slightly varying overall conformations, all having *trans* conformations with respect to the C=N bond. In the crystal, the packing features N—H⋯N hydrogen bonds, which connect mol­ecules into chains extending along the *c*-axis direction, inter­linked by C—H⋯π inter­actions (minimum H⋯*Cg* = 2.65 Å) into sheets lying parallel to (001).

## Abstract 2

The asymmetric unit of the title Schiff base contains three independent but conformationally similar mol­ecules that are linked in the crystal through inter­molecular N—H⋯N hydrogen bonds and C—H⋯π inter­actions, forming chains lying parallel to the *c*-axis direction.

## Chemical context   

Anthracene derivatives have been widely used in the field of anion recognition, metal ion fluorescent sensors, as well as *p*H sensors (Gunnlaugsson *et al.*, 2003 ▸; Chen & Chen, 2004 ▸; Kim & Yoon, 2002 ▸; Bernhardt *et al.* 2001 ▸) because of their excellent photophysical properties and high fluorescence. The crystal structures of several anthracene derivatives have been reported previously for supra­molecular photochemistry (Akiba *et al.*, 1999 ▸; Yuan *et al.*, 2004 ▸; Yamashita *et al.*, 2005 ▸). As part of our ongoing studies of Schiff bases (Faizi *et al.*, 2016 ▸), we report herein on the synthesis and crystal structure of the title compound, (*E*)-*N* ^1^-[(anthracen-9-yl)methyl­idene]-*N* ^4^-phenyl­benzene-1,4-di­amine, obtained from the condensation of 9-anthracenecarboxaldehyde with *N*-phenyl-*p*-phenyl­enedi­amine.
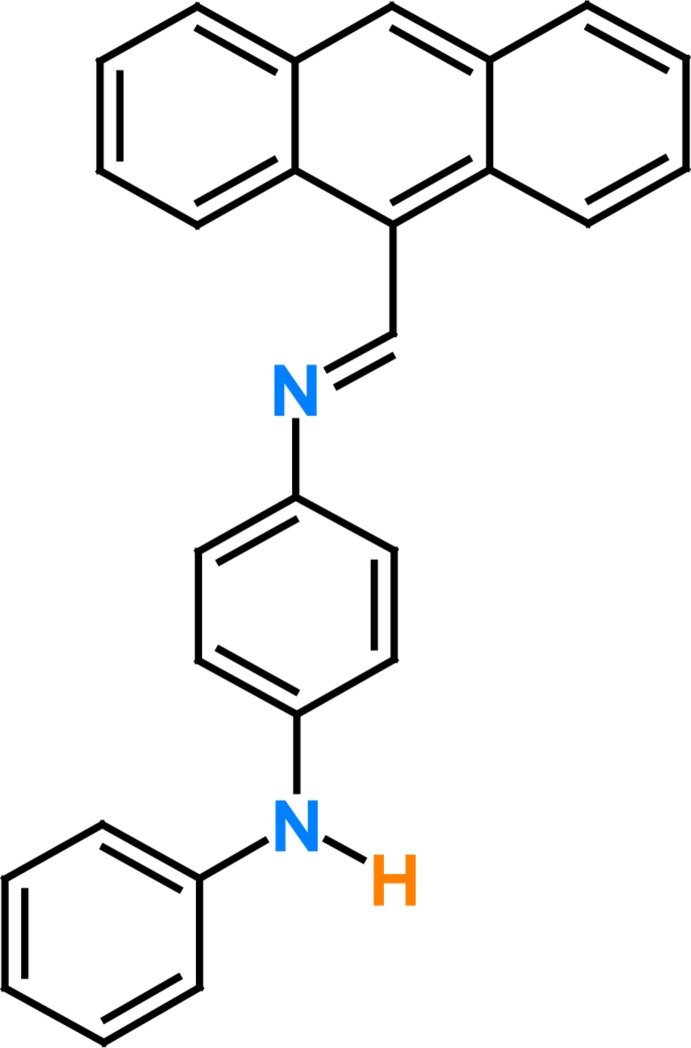


## Structural commentary   

**Figure 1:**
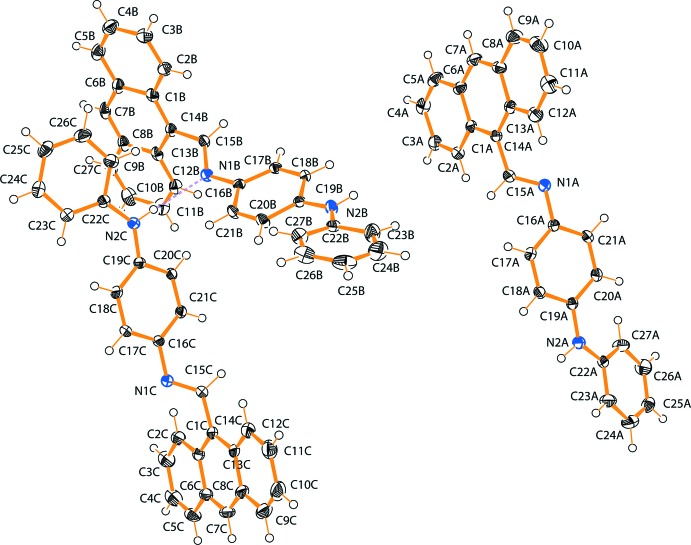
A view of the three independent mol­ecules (*A*, *B* and *C*) in the asymmetric unit of the title compound with the atom-labelling scheme and 40% probability displacement ellipsoids, showing the C—H⋯N inter­action between molecules *B* and *C* as a dashed line.

The title compound, crystallizes with three independent mol­ecules (*A*, *B* and *C*) in the asymmetric unit (Fig. 1 ▸). Mol­ecules *B* and *C* are linked by an N—H ⋯N hydrogen bond and a C—H⋯π inter­action, while mol­ecule *A* forms a C—H⋯π inter­action with mol­ecule *B*, as well as an N—H⋯N hydrogen bond and a C—H⋯π inter­action with a symmetry-generated *A* mol­ecule. An intra­molecular C—H⋯N hydrogen bond occurs in each mol­ecule (Table 1 ▸). There is a slight variation (within 3σ) in the bond lengths and angles of the three independent mol­ecules. All three mol­ecules have *trans* conformations. The central C=N (C15—N1) bond lengths are 1.277 (2), 1.276 (2) and 1.271 (2) Å for mol­ecules *A*, *B* and *C*, respectively. These are close to the literature value of 1.279 Å for C*sp* ^2^=N*sp* ^2^ bonds (Fritsky *et al.*, 2004 ▸; Penkova *et al.*, 2010 ▸). The C14—C15 bond lengths between the anthracene moiety and the central C=N bond in *A*, *B* and *C* are 1.474 (3), 1.472 (3) and 1.476 (3) Å, respectively. The comparative N1—C16 bonds connecting the central benzene ring to the central C=N bond in *A*, *B* and *C* are 1.422 (2), 1.419 (2) and 1.420 (2) Å, respectively. The C14—C15—N1—C16, torsion angles for the –C—C—N—C– bridge groups are −178.47 (17)° (for *A*), −176.35 (17)° (for *B*) and 178.31 (17)° (for *C*). The comparative dihedral angles between the anthracene ring system of the mol­ecule (defined by C1–C14) and the benzene and phenyl rings (defined by C16–C21 and C22–C27) and between the benzene and phenyl rings, respectively, are 82.68 (4), 73.76 (5) and 25.63 (11)° in *A*, 80.10 (4), 78.82 (5) and 22.56 (11)° (in *B*) and 85.02 (5), 81.66 (5) and 16.25 (11)° (in *C*).

## Supra­molecular features   

**Figure 2:**
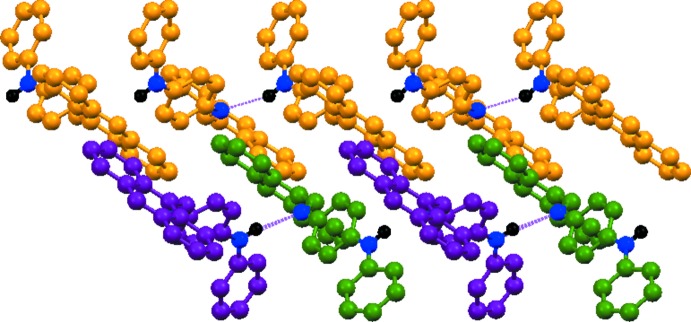
A view of the hydrogen-bonded chains propagating in [001]. Hydrogen bonds are shown as dashed lines; see Table 1 ▸ for details.

**Figure 3:**
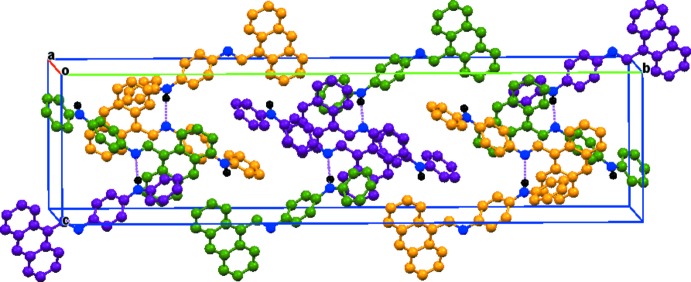
A view along the *a* axis of the crystal packing of the title compound. The hydrogen bonds are shown as dashed lines.

In the crystal, the mol­ecules are connected by N—H⋯N hydrogen bonds that result in separate –*A*–*A*–*A*–*A*– and –*B*–*C*–*B*–*C*– chains, which both propagate in [001] (Table 1 ▸ and Fig. 2 ▸). The chains are linked *via* C—H⋯π inter­actions between the phenyl and central benzene rings and those of the anthracene moiety groups of neighbouring mol­ecules [minimum C17*A*—H⋯*Cg*(C1*C*–C14*C*) = 2.65 Å; C—H⋯*Cg* = 154°], forming layers lying parallel to (001) (Fig. 3 ▸, Table 1 ▸).

## Database survey   

A search of the Cambridge Structural Database (Version 5.36; last update November 2014; Groom *et al.*, 2016 ▸) gave three hits for Schiff base compounds involving *N*-phenyl-*p*-phenyl­enedi­amine. Of these three compounds, *N*1-phenyl-*N*-4-(quinolin-2-yl­methyl­ene)benzene-1,4-di­amine {synonym: *N*-phenyl-4-[(quinolin-2-yl­methyl­ene)amino]-aniline; WOJJIQ (Faizi *et al.*, 2014 ▸] is the most similar to the title compound, with dihedral angles between quinoline ring system (r.m.s. deviation = 0.027 Å) and the central benzene and terminal phenyl rings of 44.72 (7) and 9.02 (4)°, respectively. Another similar structure crystal is that of *N*1-phenyl-*N*4-[(*E*)-(pyren-1-yl)-methyl­idene]benzene-1,4-di­amine (Faizi & Prisyazhnaya, 2015 ▸), which has dihedral angles between the pyrenyl ring system (r.m.s. deviation = 0.027 Å) and the central and terminal benzene rings of 43.43 (9) and 29.33 (7)°, respectively. Some similar ligands have been used as dual chemosensors for the detection of Cu^2+^and Hg^2+^ ions (Udhayakumari & Velmathi, 2015 ▸) but their crystal structures have not been reported.

## Synthesis and crystallization   

80 mg (0.435 mmol) of *N*-phenyl-*p*-phenyl­enedi­amine were dissolved in 10 ml of absolute ethanol. To this solution, 89 mg (0.434 mmol) of 9-anthracenecarboxaldehyde in 5 ml of absolute ethanol was added dropwise under stirring. The mixture was stirred for 10 min, two drops of glacial acetic acid were then added and the mixture was further refluxed for 2h. The resulting yellow precipitate was recovered by filtration, washed several times with small portions of ice-cold ethanol and then with diethyl ether to give 140 mg (87%) of the title compound. Dark-yellow block-like crystals suitable for X-ray analysis were obtained within 3 days by slow evaporation of a solution in MeOH.

## Refinement   

Crystal data, data collection and structure refinement details are summarized in Table 2 ▸. The N-bound H atoms were located in a difference Fourier map. Their positional and isotropic thermal parameters were included in further stages of the refinement. All C-bound H atoms were positioned geometrically and refined using a riding model with C—H = 0.93–0.97 Å and with *U*_iso_(H)= 1.2–1.5*U*_eq_(C).

## Supplementary Material

Crystal structure: contains datablock(s) I. DOI: 10.1107/S2056989016020612/zs2373sup1.cif

Structure factors: contains datablock(s) I. DOI: 10.1107/S2056989016020612/zs2373Isup2.hkl

Supporting information file. DOI: 10.1107/S2056989016020612/zs2373Isup3.cml

CCDC reference: 1524849

Additional supporting information:  crystallographic information; 3D view; checkCIF report

## Figures and Tables

**Table 1:** Hydrogen-bond geometry (Å, °) *Cg*2, *Cg*4, *Cg*10,*Cg*11, *Cg*12, *Cg*13,*Cg*18, *Cg*20 and *Cg*21are the centroids of the C1*A*–C14*A*, C16*A*–C21*A*, C1*B*–C14*B*, C8*B*–C13*B*, C16*B*–C21*B*, C22*B*–C27*B*, C1*C*–C14*C*, C22*C*–C27*C* and C1*C*–C8*C*rings, respectively.

*D*—H⋯*A*	*D*—H	H⋯*A*	*D*⋯*A*	*D*—H⋯*A*
C12*A*—H9*A*⋯N1*A*	0.93	2.44	2.989 (3)	118
C12*B*—H9*B*⋯N1*B*	0.93	2.47	3.006 (3)	117
C2*C*—H1*C*⋯N1*C*	0.93	2.51	3.032 (3)	116
N2*A*—H20*A*⋯N1*A* ^i^	0.92 (2)	2.28 (2)	3.147 (2)	158.4 (18)
N2*B*—H20*B*⋯N1*C* ^ii^	0.88 (2)	2.19 (2)	3.056 (2)	166.2 (19)
N2*C*—H20*C*⋯N1*B*	0.90 (2)	2.22 (2)	3.094 (2)	163.2 (17)
C2*A*—H1*A*⋯*Cg*13^iii^	0.93	2.93	3.7248 (3)	144
C2*B*—H1*B*⋯*Cg*21^iv^	0.93	2.91	3.6227 (3)	134
C4*A*—H3*A*⋯*Cg*21^v^	0.93	2.75	3.6728 (3)	175
C10*A*—H7*A*⋯*Cg*12^ii^	0.93	2.80	3.5075 (3)	134
C17*A*—H11*A*⋯*Cg*18^ii^	0.93	2.65	3.5119 (3)	154
C17*B*—H11*B*⋯*Cg*10^v^	0.93	2.79	3.6243 (3)	150
C17*B*—H11*B*⋯*Cg*11^v^	0.93	2.89	3.7248 (3)	150
C21*C*—H13*C*⋯*Cg*2^vi^	0.93	2.86	3.7513 (3)	162
C23*A*—H15*A*⋯*Cg*2^i^	0.93	2.85	3.7253 (3)	157
C23*B*—H15*B*⋯*Cg*18^ii^	0.93	2.87	3.7057 (3)	149
C25*A*—H17*A*⋯*Cg*20^vii^	0.93	2.96	3.6262 (3)	130
C25*B*—H17*B*⋯*Cg*4^viii^	0.93	3.00	3.6476 (3)	128
C25*C*—H17*C*⋯*Cg*12^iv^	0.93	2.84	3.6263 (3)	143

Symmetry codes: (i) 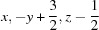; (ii) 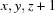; (iii) 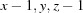; (iv) 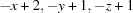; (v) 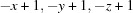; (vi) 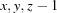; (vii) 
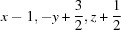
; (viii) 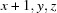.

**Table 2:** Experimental details

Crystal data	
Chemical formula	C_27_H_20_N_2_
*M*_r_	372.45
Crystal system, space group	Monoclinic, *P*2_1_/*c*
Temperature (K)	100
*a*, *b*, *c* (Å)	11.1554 (8), 45.224 (3), 11.5856 (8)
β (°)	96.645 (2)
*V* (Å^3^)	5805.5 (7)
*Z*	12
Radiation type	Mo *K*α
μ (mm^−1^)	0.08
Crystal size (mm)	0.20 × 0.15 × 0.10

Data collection	
Diffractometer	BRUKER SMART APEX CCD
Absorption correction	Multi-scan (*SADABS*; Bruker, 2003 ▸)
*T*_min_, *T*_max_	0.944, 0.981
No. of measured, independent and observed [*I* > 2σ(*I*)] reflections	70261, 10276, 6957
*R*_int_	0.066
(sin θ/λ)_max_ (Å^−1^)	0.596

Refinement	
*R*[*F* ^2^ > 2σ(*F* ^2^)], *wR*(*F* ^2^), *S*	0.052, 0.121, 1.04
No. of reflections	10276
No. of parameters	796
H-atom treatment	H atoms treated by a mixture of independent and constrained refinement
Δρ_max_, Δρ_min_ (e Å^−3^)	0.18, −0.28

Computer programs: *SMART* and *SAINT* (Bruker, 2003 ▸), *SIR97* (Altomare *et al.*, 1999 ▸), *SHELXL2014* (Sheldrick, 2015 ▸) and *DIAMOND* (Brandenberg & Putz, 2006 ▸).

## supplementary crystallographic information

### Crystal data

**Table:**

C_27_H_20_N_2_	*F*(000) = 2352
*M**_r_* = 372.45	*D*_x_ = 1.278 Mg m^−^^3^
Monoclinic, *P*2_1_/*c*	Mo *K*α radiation, λ = 0.71073 Å
*a* = 11.1554 (8) Å	Cell parameters from 9870 reflections
*b* = 45.224 (3) Å	θ = 2.7–27.3°
*c* = 11.5856 (8) Å	µ = 0.08 mm^−^^1^
β = 96.645 (2)°	*T* = 100 K
*V* = 5805.5 (7) Å^3^	Block, yellow
*Z* = 12	0.20 × 0.15 × 0.10 mm

### Data collection

**Table:**

BRUKER SMART APEX CCD diffractometer	10276 independent reflections
Radiation source: fine-focus sealed tube	6957 reflections with *I* > 2σ(*I*)
Graphite monochromator	*R*_int_ = 0.066
ω scans	θ_max_ = 25.1°, θ_min_ = 2.1°
Absorption correction: multi-scan (SADABS; Bruker, 2003)	*h* = −13→13
*T*_min_ = 0.944, *T*_max_ = 0.981	*k* = −53→53
70261 measured reflections	*l* = −13→13

### Refinement

**Table:**

Refinement on *F*^2^	0 restraints
Least-squares matrix: full	Hydrogen site location: mixed
*R*[*F*^2^ > 2σ(*F*^2^)] = 0.052	H atoms treated by a mixture of independent and constrained refinement
*wR*(*F*^2^) = 0.121	*w* = 1/[σ^2^(*F*_o_^2^) + (0.0468*P*)^2^ + 1.868*P*] where *P* = (*F*_o_^2^ + 2*F*_c_^2^)/3
*S* = 1.04	(Δ/σ)_max_ = 0.001
10276 reflections	Δρ_max_ = 0.18 e Å^−^^3^
796 parameters	Δρ_min_ = −0.28 e Å^−^^3^

### Special details

**Table:**

Geometry. All esds (except the esd in the dihedral angle between two l.s. planes) are estimated using the full covariance matrix. The cell esds are taken into account individually in the estimation of esds in distances, angles and torsion angles; correlations between esds in cell parameters are only used when they are defined by crystal symmetry. An approximate (isotropic) treatment of cell esds is used for estimating esds involving l.s. planes.

### Fractional atomic coordinates and isotropic or equivalent isotropic displacement parameters (Å^2^)

**Table:**

	*x*	*y*	*z*	*U*_iso_*/*U*_eq_	
C1A	0.26112 (17)	0.61937 (4)	1.02008 (17)	0.0262 (5)	
C1B	0.71492 (18)	0.45788 (4)	0.46717 (17)	0.0282 (5)	
C1C	0.55519 (18)	0.68363 (4)	−0.18917 (18)	0.0314 (5)	
C2A	0.18402 (18)	0.62145 (5)	0.91327 (17)	0.0313 (5)	
H1A	0.1713	0.6398	0.8776	0.038*	
C2B	0.78872 (19)	0.45822 (4)	0.57631 (18)	0.0327 (5)	
H1B	0.7929	0.4753	0.6211	0.039*	
C2C	0.49613 (18)	0.65789 (5)	−0.23828 (18)	0.0357 (5)	
H1C	0.5033	0.6402	−0.1971	0.043*	
C3A	0.12888 (19)	0.59724 (5)	0.86268 (19)	0.0352 (5)	
H2A	0.0798	0.5993	0.7925	0.042*	
C3B	0.8530 (2)	0.43405 (5)	0.61612 (19)	0.0393 (6)	
H2B	0.8994	0.4348	0.6881	0.047*	
C3C	0.4297 (2)	0.65861 (6)	−0.34359 (19)	0.0442 (6)	
H2C	0.3931	0.6413	−0.3737	0.053*	
C4A	0.14451 (19)	0.56901 (5)	0.9145 (2)	0.0380 (6)	
H3A	0.1077	0.5525	0.8779	0.046*	
C4B	0.8504 (2)	0.40788 (5)	0.5497 (2)	0.0418 (6)	
H3B	0.8948	0.3915	0.5779	0.050*	
C4C	0.4149 (2)	0.68491 (6)	−0.4086 (2)	0.0503 (7)	
H3C	0.3687	0.6850	−0.4808	0.060*	
C5A	0.21301 (19)	0.56617 (5)	1.0172 (2)	0.0375 (6)	
H4A	0.2215	0.5476	1.0519	0.045*	
C5B	0.7831 (2)	0.40671 (5)	0.4451 (2)	0.0385 (6)	
H4B	0.7823	0.3894	0.4017	0.046*	
C5C	0.4680 (2)	0.71001 (6)	−0.3653 (2)	0.0474 (7)	
H4C	0.4576	0.7274	−0.4083	0.057*	
C6A	0.27303 (18)	0.59094 (4)	1.07454 (18)	0.0309 (5)	
C6B	0.71353 (18)	0.43128 (4)	0.39983 (18)	0.0303 (5)	
C6C	0.53946 (19)	0.71046 (5)	−0.25565 (19)	0.0366 (6)	
C7A	0.33961 (19)	0.58833 (5)	1.18216 (19)	0.0351 (5)	
H5A	0.3449	0.5699	1.2181	0.042*	
C7B	0.64679 (18)	0.43032 (4)	0.29127 (18)	0.0331 (5)	
H5B	0.6468	0.4130	0.2481	0.040*	
C7C	0.5933 (2)	0.73624 (5)	−0.2093 (2)	0.0435 (6)	
H5C	0.5837	0.7535	−0.2528	0.052*	
C8A	0.39895 (18)	0.61213 (5)	1.23881 (18)	0.0318 (5)	
C8B	0.57997 (18)	0.45425 (4)	0.24448 (17)	0.0298 (5)	
C8C	0.6606 (2)	0.73722 (5)	−0.1012 (2)	0.0392 (6)	
C9A	0.4676 (2)	0.60887 (5)	1.34924 (19)	0.0416 (6)	
H6A	0.4707	0.5905	1.3858	0.050*	
C9B	0.5082 (2)	0.45245 (5)	0.13519 (18)	0.0382 (6)	
H6B	0.5086	0.4351	0.0922	0.046*	
C9C	0.7169 (2)	0.76360 (5)	−0.0555 (3)	0.0531 (7)	
H6C	0.7056	0.7810	−0.0983	0.064*	
C10A	0.5281 (2)	0.63167 (6)	1.4019 (2)	0.0467 (6)	
H7A	0.5723	0.6291	1.4743	0.056*	
C10B	0.4397 (2)	0.47522 (5)	0.09266 (19)	0.0431 (6)	
H7B	0.3930	0.4736	0.0211	0.052*	
C10C	0.7862 (3)	0.76430 (5)	0.0479 (3)	0.0566 (8)	
H7C	0.8209	0.7820	0.0760	0.068*	
C11A	0.52493 (19)	0.65969 (5)	1.34739 (19)	0.0428 (6)	
H8A	0.5686	0.6753	1.3838	0.051*	
C11B	0.4388 (2)	0.50170 (5)	0.15699 (19)	0.0408 (6)	
H8B	0.3904	0.5173	0.1275	0.049*	
C11C	0.8057 (2)	0.73793 (5)	0.1131 (2)	0.0501 (7)	
H8C	0.8543	0.7383	0.1839	0.060*	
C12A	0.45909 (18)	0.66419 (5)	1.24274 (18)	0.0339 (5)	
H9A	0.4579	0.6828	1.2088	0.041*	
C12B	0.50720 (18)	0.50484 (5)	0.26098 (18)	0.0314 (5)	
H9B	0.5058	0.5227	0.3009	0.038*	
C12C	0.7541 (2)	0.71209 (5)	0.07366 (19)	0.0409 (6)	
H9C	0.7688	0.6950	0.1177	0.049*	
C13A	0.39169 (17)	0.64070 (4)	1.18408 (17)	0.0271 (5)	
C13B	0.58098 (17)	0.48128 (4)	0.30983 (17)	0.0266 (5)	
C13C	0.67808 (19)	0.71060 (4)	−0.03370 (18)	0.0328 (5)	
C14A	0.32106 (17)	0.64399 (4)	1.07609 (16)	0.0252 (5)	
C14B	0.65076 (17)	0.48293 (4)	0.41958 (17)	0.0249 (5)	
C14C	0.62503 (18)	0.68413 (4)	−0.07964 (18)	0.0298 (5)	
C15A	0.30094 (17)	0.67319 (4)	1.02085 (17)	0.0262 (5)	
H10A	0.3043	0.6748	0.9412	0.031*	
C15B	0.66532 (17)	0.51095 (4)	0.48428 (16)	0.0248 (5)	
H10B	0.6577	0.5111	0.5634	0.030*	
C15C	0.65310 (18)	0.65659 (4)	−0.01366 (17)	0.0275 (5)	
H10C	0.6407	0.6557	0.0643	0.033*	
C16A	0.25826 (17)	0.72330 (4)	1.01442 (16)	0.0233 (4)	
C16B	0.70840 (16)	0.56121 (4)	0.50147 (15)	0.0211 (4)	
C16C	0.72269 (17)	0.60827 (4)	0.00646 (15)	0.0215 (4)	
C17A	0.32184 (17)	0.73145 (4)	0.92280 (16)	0.0244 (4)	
H11A	0.3854	0.7197	0.9041	0.029*	
C17B	0.65393 (16)	0.56740 (4)	0.60083 (15)	0.0225 (4)	
H11B	0.5946	0.5548	0.6227	0.027*	
C17C	0.82786 (18)	0.59356 (4)	−0.00799 (16)	0.0267 (5)	
H11C	0.8743	0.5999	−0.0649	0.032*	
C18A	0.29143 (17)	0.75671 (4)	0.85951 (16)	0.0240 (4)	
H12A	0.3351	0.7619	0.7988	0.029*	
C18B	0.68698 (16)	0.59196 (4)	0.66704 (16)	0.0226 (4)	
H12B	0.6498	0.5956	0.7334	0.027*	
C18C	0.86612 (17)	0.56949 (4)	0.06038 (16)	0.0266 (5)	
H12C	0.9396	0.5606	0.0515	0.032*	
C19A	0.19624 (17)	0.77470 (4)	0.88491 (16)	0.0220 (4)	
C19B	0.77522 (16)	0.61147 (4)	0.63675 (15)	0.0210 (4)	
C19C	0.79554 (17)	0.55846 (4)	0.14235 (15)	0.0211 (4)	
C20A	0.13906 (18)	0.76750 (4)	0.98216 (16)	0.0289 (5)	
H13A	0.0791	0.7798	1.0045	0.035*	
C20B	0.82151 (18)	0.60648 (4)	0.53181 (16)	0.0287 (5)	
H13B	0.8753	0.6200	0.5058	0.034*	
C20C	0.68619 (17)	0.57274 (4)	0.15263 (16)	0.0231 (4)	
H14C	0.6362	0.5655	0.2050	0.028*	
C21A	0.17071 (18)	0.74221 (4)	1.04569 (16)	0.0287 (5)	
H14A	0.1322	0.7379	1.1107	0.034*	
C21B	0.78838 (18)	0.58171 (4)	0.46604 (16)	0.0278 (5)	
H14B	0.8206	0.5788	0.3964	0.033*	
C21C	0.65063 (17)	0.59727 (4)	0.08713 (15)	0.0236 (4)	
H13C	0.5781	0.6066	0.0968	0.028*	
C22A	0.06858 (17)	0.81786 (4)	0.80373 (16)	0.0238 (4)	
C22B	0.90242 (17)	0.65480 (4)	0.71494 (16)	0.0238 (4)	
C22C	0.92058 (17)	0.51468 (4)	0.22658 (16)	0.0235 (4)	
C23A	0.0768 (2)	0.84370 (5)	0.74049 (19)	0.0386 (6)	
H15A	0.1485	0.8480	0.7103	0.046*	
C23B	0.8996 (2)	0.67904 (5)	0.78831 (19)	0.0408 (6)	
H15B	0.8339	0.6816	0.8298	0.049*	
C23C	1.00215 (18)	0.51024 (4)	0.14642 (18)	0.0321 (5)	
H15C	0.9970	0.5217	0.0795	0.038*	
C24A	−0.0180 (2)	0.86307 (5)	0.7212 (2)	0.0432 (6)	
H16A	−0.0101	0.8800	0.6773	0.052*	
C24B	0.9923 (2)	0.69923 (5)	0.8007 (2)	0.0508 (7)	
H16B	0.9884	0.7152	0.8506	0.061*	
C24C	1.09106 (19)	0.48884 (4)	0.16520 (18)	0.0346 (5)	
H16C	1.1460	0.4865	0.1113	0.042*	
C25A	−0.1242 (2)	0.85767 (5)	0.76608 (18)	0.0357 (5)	
H17A	−0.1883	0.8709	0.7543	0.043*	
C25B	1.0897 (2)	0.69612 (5)	0.7408 (2)	0.0461 (6)	
H17B	1.1516	0.7100	0.7483	0.055*	
C25C	1.09985 (19)	0.47100 (5)	0.26146 (19)	0.0375 (6)	
H17C	1.1585	0.4563	0.2721	0.045*	
C26A	−0.1336 (2)	0.83227 (5)	0.8286 (2)	0.0419 (6)	
H18A	−0.2051	0.8284	0.8599	0.050*	
C26B	1.0947 (2)	0.67210 (5)	0.66918 (19)	0.0427 (6)	
H18B	1.1611	0.6697	0.6284	0.051*	
C26C	1.0197 (2)	0.47527 (5)	0.34235 (19)	0.0402 (6)	
H18C	1.0246	0.4634	0.4084	0.048*	
C27A	−0.03963 (19)	0.81222 (5)	0.84664 (18)	0.0361 (5)	
H19A	−0.0493	0.7949	0.8878	0.043*	
C27B	1.00284 (18)	0.65145 (5)	0.65651 (18)	0.0331 (5)	
H19B	1.0086	0.6352	0.6084	0.040*	
C27C	0.93266 (19)	0.49686 (4)	0.32602 (17)	0.0332 (5)	
H19C	0.8807	0.4997	0.3823	0.040*	
N1A	0.27915 (14)	0.69645 (3)	1.07744 (13)	0.0248 (4)	
N1B	0.68818 (13)	0.53510 (3)	0.43417 (13)	0.0230 (4)	
N1C	0.69395 (14)	0.63399 (3)	−0.06137 (13)	0.0248 (4)	
N2A	0.16966 (15)	0.79942 (3)	0.81534 (14)	0.0268 (4)	
N2B	0.80552 (15)	0.63521 (4)	0.70989 (14)	0.0263 (4)	
N2C	0.82550 (15)	0.53481 (3)	0.21699 (14)	0.0254 (4)	
H20A	0.2198 (19)	0.8020 (5)	0.7589 (19)	0.044 (7)*	
H20B	0.7628 (19)	0.6364 (4)	0.7690 (18)	0.038 (6)*	
H20C	0.7737 (17)	0.5329 (4)	0.2703 (17)	0.027 (6)*	

### Atomic displacement parameters (Å^2^)

**Table:**

	*U*^11^	*U*^22^	*U*^33^	*U*^12^	*U*^13^	*U*^23^
C1A	0.0274 (11)	0.0251 (11)	0.0284 (11)	0.0057 (9)	0.0130 (9)	0.0017 (9)
C1B	0.0298 (12)	0.0254 (11)	0.0321 (12)	−0.0082 (9)	0.0153 (10)	0.0008 (9)
C1C	0.0297 (12)	0.0325 (12)	0.0349 (12)	0.0139 (10)	0.0156 (10)	0.0094 (10)
C2A	0.0385 (13)	0.0282 (12)	0.0285 (12)	0.0030 (10)	0.0092 (10)	0.0019 (9)
C2B	0.0411 (13)	0.0237 (11)	0.0346 (13)	−0.0042 (10)	0.0094 (11)	0.0004 (10)
C2C	0.0297 (12)	0.0417 (14)	0.0367 (13)	0.0101 (10)	0.0078 (10)	0.0099 (11)
C3A	0.0375 (13)	0.0336 (13)	0.0356 (12)	−0.0017 (10)	0.0092 (10)	−0.0045 (10)
C3B	0.0458 (14)	0.0340 (13)	0.0391 (13)	−0.0011 (11)	0.0088 (11)	0.0055 (11)
C3C	0.0318 (13)	0.0597 (16)	0.0409 (14)	0.0071 (12)	0.0027 (11)	0.0084 (12)
C4A	0.0332 (12)	0.0330 (13)	0.0493 (15)	−0.0052 (10)	0.0112 (12)	−0.0053 (11)
C4B	0.0422 (14)	0.0296 (13)	0.0554 (16)	0.0042 (11)	0.0137 (13)	0.0075 (12)
C4C	0.0355 (14)	0.076 (2)	0.0398 (14)	0.0160 (14)	0.0055 (11)	0.0158 (14)
C5A	0.0339 (12)	0.0231 (12)	0.0579 (16)	−0.0005 (10)	0.0161 (12)	0.0050 (11)
C5B	0.0386 (13)	0.0252 (12)	0.0544 (16)	−0.0025 (10)	0.0162 (12)	−0.0051 (11)
C5C	0.0414 (14)	0.0595 (17)	0.0442 (15)	0.0261 (13)	0.0170 (12)	0.0282 (13)
C6A	0.0268 (11)	0.0275 (12)	0.0409 (13)	0.0077 (9)	0.0147 (10)	0.0067 (10)
C6B	0.0304 (11)	0.0252 (12)	0.0381 (13)	−0.0060 (10)	0.0159 (10)	−0.0042 (10)
C6C	0.0348 (13)	0.0364 (13)	0.0422 (14)	0.0162 (11)	0.0202 (11)	0.0156 (11)
C7A	0.0334 (12)	0.0289 (12)	0.0459 (14)	0.0082 (10)	0.0174 (11)	0.0130 (11)
C7B	0.0344 (12)	0.0258 (12)	0.0426 (14)	−0.0098 (10)	0.0189 (11)	−0.0139 (10)
C7C	0.0447 (14)	0.0351 (14)	0.0564 (16)	0.0174 (11)	0.0299 (13)	0.0252 (12)
C8A	0.0259 (11)	0.0403 (13)	0.0312 (12)	0.0144 (10)	0.0112 (10)	0.0121 (10)
C8B	0.0273 (11)	0.0320 (12)	0.0330 (12)	−0.0104 (10)	0.0157 (10)	−0.0080 (10)
C8C	0.0429 (14)	0.0253 (12)	0.0550 (16)	0.0077 (10)	0.0289 (12)	0.0088 (11)
C9A	0.0387 (14)	0.0470 (15)	0.0396 (14)	0.0156 (12)	0.0062 (11)	0.0124 (12)
C9B	0.0378 (13)	0.0424 (14)	0.0358 (13)	−0.0136 (11)	0.0104 (11)	−0.0163 (11)
C9C	0.0625 (18)	0.0279 (14)	0.076 (2)	0.0044 (12)	0.0374 (16)	0.0078 (13)
C10A	0.0365 (14)	0.0704 (18)	0.0319 (13)	0.0195 (13)	−0.0019 (11)	0.0107 (13)
C10B	0.0377 (14)	0.0572 (17)	0.0332 (13)	−0.0104 (12)	−0.0005 (11)	−0.0129 (12)
C10C	0.0690 (19)	0.0333 (15)	0.075 (2)	−0.0108 (13)	0.0412 (17)	−0.0121 (14)
C11A	0.0284 (12)	0.0577 (16)	0.0413 (14)	0.0047 (11)	0.0002 (11)	−0.0017 (12)
C11B	0.0360 (13)	0.0451 (14)	0.0399 (14)	−0.0018 (11)	−0.0014 (11)	−0.0065 (11)
C11C	0.0623 (17)	0.0406 (15)	0.0514 (15)	−0.0098 (13)	0.0243 (13)	−0.0112 (13)
C12A	0.0272 (12)	0.0412 (13)	0.0334 (12)	0.0077 (10)	0.0035 (10)	0.0021 (10)
C12B	0.0301 (12)	0.0309 (12)	0.0336 (12)	−0.0051 (10)	0.0059 (10)	−0.0052 (10)
C12C	0.0547 (15)	0.0297 (13)	0.0417 (14)	−0.0007 (11)	0.0202 (12)	−0.0016 (11)
C13A	0.0231 (11)	0.0324 (12)	0.0275 (11)	0.0084 (9)	0.0096 (9)	0.0021 (9)
C13B	0.0259 (11)	0.0271 (11)	0.0290 (11)	−0.0100 (9)	0.0126 (9)	−0.0046 (9)
C13C	0.0407 (13)	0.0239 (12)	0.0384 (13)	0.0075 (10)	0.0233 (11)	0.0019 (10)
C14A	0.0260 (11)	0.0261 (11)	0.0253 (11)	0.0069 (9)	0.0101 (9)	0.0038 (9)
C14B	0.0263 (11)	0.0231 (11)	0.0275 (11)	−0.0068 (9)	0.0121 (9)	−0.0031 (9)
C14C	0.0347 (12)	0.0252 (12)	0.0326 (12)	0.0094 (10)	0.0170 (10)	0.0044 (9)
C15A	0.0286 (11)	0.0272 (11)	0.0237 (11)	0.0017 (9)	0.0071 (9)	0.0014 (9)
C15B	0.0280 (11)	0.0264 (11)	0.0212 (10)	−0.0021 (9)	0.0078 (9)	−0.0015 (9)
C15C	0.0351 (12)	0.0258 (11)	0.0230 (11)	0.0029 (9)	0.0094 (9)	0.0024 (9)
C16A	0.0278 (11)	0.0212 (10)	0.0204 (10)	0.0001 (9)	0.0014 (9)	−0.0015 (8)
C16B	0.0227 (10)	0.0203 (10)	0.0194 (10)	0.0003 (8)	−0.0013 (8)	0.0010 (8)
C16C	0.0282 (11)	0.0198 (10)	0.0165 (10)	0.0005 (9)	0.0020 (9)	−0.0005 (8)
C17A	0.0242 (11)	0.0233 (11)	0.0260 (11)	0.0022 (9)	0.0038 (9)	−0.0020 (9)
C17B	0.0214 (10)	0.0212 (11)	0.0251 (11)	−0.0020 (8)	0.0041 (9)	0.0037 (9)
C17C	0.0326 (12)	0.0253 (11)	0.0242 (11)	0.0017 (9)	0.0116 (9)	0.0044 (9)
C18A	0.0269 (11)	0.0243 (11)	0.0216 (10)	−0.0027 (9)	0.0057 (9)	−0.0017 (9)
C18B	0.0250 (11)	0.0233 (11)	0.0207 (10)	0.0022 (9)	0.0080 (9)	−0.0004 (8)
C18C	0.0248 (11)	0.0257 (11)	0.0307 (11)	0.0045 (9)	0.0096 (9)	0.0027 (9)
C19A	0.0252 (11)	0.0199 (10)	0.0200 (10)	−0.0020 (9)	−0.0006 (8)	−0.0027 (8)
C19B	0.0233 (11)	0.0191 (10)	0.0202 (10)	0.0005 (8)	0.0011 (8)	0.0010 (8)
C19C	0.0263 (11)	0.0191 (10)	0.0173 (10)	−0.0021 (8)	0.0001 (8)	−0.0016 (8)
C20A	0.0327 (12)	0.0291 (12)	0.0265 (11)	0.0082 (9)	0.0095 (10)	−0.0006 (9)
C20B	0.0328 (12)	0.0292 (12)	0.0256 (11)	−0.0125 (10)	0.0102 (9)	−0.0023 (9)
C20C	0.0273 (11)	0.0222 (10)	0.0207 (10)	−0.0016 (9)	0.0075 (9)	−0.0011 (8)
C21A	0.0359 (12)	0.0312 (12)	0.0205 (11)	0.0055 (10)	0.0093 (9)	0.0022 (9)
C21B	0.0358 (12)	0.0300 (12)	0.0192 (10)	−0.0062 (10)	0.0097 (9)	−0.0027 (9)
C21C	0.0229 (10)	0.0247 (11)	0.0235 (10)	0.0041 (9)	0.0040 (9)	−0.0010 (9)
C22A	0.0287 (11)	0.0238 (11)	0.0186 (10)	0.0020 (9)	0.0016 (9)	−0.0018 (8)
C22B	0.0271 (11)	0.0214 (11)	0.0218 (10)	−0.0026 (9)	−0.0012 (9)	0.0021 (9)
C22C	0.0246 (11)	0.0207 (10)	0.0248 (11)	−0.0021 (9)	0.0009 (9)	−0.0019 (9)
C23A	0.0405 (14)	0.0350 (13)	0.0420 (14)	0.0050 (11)	0.0122 (11)	0.0127 (11)
C23B	0.0465 (14)	0.0348 (13)	0.0421 (14)	−0.0084 (11)	0.0092 (11)	−0.0112 (11)
C23C	0.0387 (13)	0.0277 (12)	0.0308 (12)	0.0060 (10)	0.0085 (10)	0.0028 (10)
C24A	0.0505 (15)	0.0333 (13)	0.0451 (14)	0.0077 (12)	0.0029 (12)	0.0119 (11)
C24B	0.0685 (18)	0.0318 (14)	0.0498 (16)	−0.0171 (13)	−0.0022 (14)	−0.0102 (12)
C24C	0.0323 (12)	0.0340 (13)	0.0380 (13)	0.0055 (10)	0.0058 (10)	−0.0069 (11)
C25A	0.0377 (13)	0.0317 (13)	0.0351 (13)	0.0123 (10)	−0.0074 (11)	−0.0046 (10)
C25B	0.0501 (16)	0.0398 (14)	0.0436 (14)	−0.0243 (12)	−0.0147 (13)	0.0132 (12)
C25C	0.0334 (13)	0.0348 (13)	0.0416 (14)	0.0108 (10)	−0.0068 (11)	−0.0041 (11)
C26A	0.0298 (12)	0.0488 (15)	0.0480 (15)	0.0075 (11)	0.0080 (11)	0.0059 (12)
C26B	0.0314 (13)	0.0545 (16)	0.0411 (14)	−0.0125 (12)	−0.0004 (11)	0.0079 (12)
C26C	0.0433 (14)	0.0365 (13)	0.0390 (13)	0.0102 (11)	−0.0030 (11)	0.0096 (11)
C27A	0.0333 (12)	0.0334 (13)	0.0423 (13)	0.0032 (10)	0.0071 (11)	0.0139 (10)
C27B	0.0290 (12)	0.0363 (13)	0.0334 (12)	−0.0065 (10)	0.0014 (10)	−0.0030 (10)
C27C	0.0341 (12)	0.0347 (12)	0.0312 (12)	0.0025 (10)	0.0063 (10)	0.0052 (10)
N1A	0.0264 (9)	0.0244 (9)	0.0239 (9)	0.0050 (7)	0.0041 (7)	0.0008 (8)
N1B	0.0236 (9)	0.0229 (9)	0.0228 (9)	−0.0041 (7)	0.0042 (7)	−0.0015 (7)
N1C	0.0283 (9)	0.0237 (9)	0.0229 (9)	0.0037 (8)	0.0055 (7)	0.0018 (7)
N2A	0.0292 (10)	0.0254 (10)	0.0271 (9)	0.0047 (8)	0.0088 (8)	0.0032 (8)
N2B	0.0287 (10)	0.0262 (10)	0.0251 (9)	−0.0058 (8)	0.0085 (8)	−0.0062 (8)
N2C	0.0287 (10)	0.0248 (9)	0.0243 (9)	0.0044 (8)	0.0093 (8)	0.0051 (8)

### Geometric parameters (Å, º)

**Table:**

C1A—C14A	1.417 (3)	C15B—N1B	1.276 (2)
C1A—C2A	1.426 (3)	C15B—H10B	0.9300
C1A—C6A	1.432 (3)	C15C—N1C	1.271 (2)
C1B—C14B	1.417 (3)	C15C—H10C	0.9300
C1B—C2B	1.427 (3)	C16A—C21A	1.378 (3)
C1B—C6B	1.433 (3)	C16A—C17A	1.393 (3)
C1C—C14C	1.410 (3)	C16A—N1A	1.422 (2)
C1C—C2C	1.423 (3)	C16B—C21B	1.381 (3)
C1C—C6C	1.437 (3)	C16B—C17B	1.391 (2)
C2A—C3A	1.355 (3)	C16B—N1B	1.419 (2)
C2A—H1A	0.9300	C16C—C17C	1.376 (3)
C2B—C3B	1.359 (3)	C16C—C21C	1.393 (3)
C2B—H1B	0.9300	C16C—N1C	1.420 (2)
C2C—C3C	1.352 (3)	C17A—C18A	1.379 (3)
C2C—H1C	0.9300	C17A—H11A	0.9300
C3A—C4A	1.413 (3)	C17B—C18B	1.375 (2)
C3A—H2A	0.9300	C17B—H11B	0.9300
C3B—C4B	1.410 (3)	C17C—C18C	1.384 (3)
C3B—H2B	0.9300	C17C—H11C	0.9300
C3C—C4C	1.407 (3)	C18A—C19A	1.396 (3)
C3C—H2C	0.9300	C18A—H12A	0.9300
C4A—C5A	1.344 (3)	C18B—C19B	1.397 (3)
C4A—H3A	0.9300	C18B—H12B	0.9300
C4B—C5B	1.351 (3)	C18C—C19C	1.394 (3)
C4B—H3B	0.9300	C18C—H12C	0.9300
C4C—C5C	1.350 (3)	C19A—N2A	1.390 (2)
C4C—H3C	0.9300	C19A—C20A	1.396 (3)
C5A—C6A	1.429 (3)	C19B—N2B	1.385 (2)
C5A—H4A	0.9300	C19B—C20B	1.393 (3)
C5B—C6B	1.420 (3)	C19C—N2C	1.392 (2)
C5B—H4B	0.9300	C19C—C20C	1.397 (3)
C5C—C6C	1.419 (3)	C20A—C21A	1.383 (3)
C5C—H4C	0.9300	C20A—H13A	0.9300
C6A—C7A	1.381 (3)	C20B—C21B	1.381 (3)
C6B—C7B	1.386 (3)	C20B—H13B	0.9300
C6C—C7C	1.390 (3)	C20C—C21C	1.376 (2)
C7A—C8A	1.388 (3)	C20C—H14C	0.9300
C7A—H5A	0.9300	C21A—H14A	0.9300
C7B—C8B	1.388 (3)	C21B—H14B	0.9300
C7B—H5B	0.9300	C21C—H13C	0.9300
C7C—C8C	1.385 (3)	C22A—C27A	1.381 (3)
C7C—H5C	0.9300	C22A—C23A	1.388 (3)
C8A—C9A	1.421 (3)	C22A—N2A	1.396 (2)
C8A—C13A	1.437 (3)	C22B—C27B	1.383 (3)
C8B—C9B	1.420 (3)	C22B—C23B	1.390 (3)
C8B—C13B	1.437 (3)	C22B—N2B	1.394 (2)
C8C—C9C	1.422 (3)	C22C—C23C	1.388 (3)
C8C—C13C	1.436 (3)	C22C—N2C	1.392 (2)
C9A—C10A	1.340 (3)	C22C—C27C	1.400 (3)
C9A—H6A	0.9300	C23A—C24A	1.371 (3)
C9B—C10B	1.341 (3)	C23A—H15A	0.9300
C9B—H6B	0.9300	C23B—C24B	1.374 (3)
C9C—C10C	1.349 (4)	C23B—H15B	0.9300
C9C—H6C	0.9300	C23C—C24C	1.385 (3)
C10A—C11A	1.414 (3)	C23C—H15C	0.9300
C10A—H7A	0.9300	C24A—C25A	1.370 (3)
C10B—C11B	1.411 (3)	C24A—H16A	0.9300
C10B—H7B	0.9300	C24B—C25B	1.363 (3)
C10C—C11C	1.415 (3)	C24B—H16B	0.9300
C10C—H7C	0.9300	C24C—C25C	1.371 (3)
C11A—C12A	1.358 (3)	C24C—H16C	0.9300
C11A—H8A	0.9300	C25A—C26A	1.369 (3)
C11B—C12B	1.357 (3)	C25A—H17A	0.9300
C11B—H8B	0.9300	C25B—C26B	1.372 (3)
C11C—C12C	1.358 (3)	C25B—H17B	0.9300
C11C—H8C	0.9300	C25C—C26C	1.382 (3)
C12A—C13A	1.428 (3)	C25C—H17C	0.9300
C12A—H9A	0.9300	C26A—C27A	1.384 (3)
C12B—C13B	1.423 (3)	C26A—H18A	0.9300
C12B—H9B	0.9300	C26B—C27B	1.382 (3)
C12C—C13C	1.424 (3)	C26B—H18B	0.9300
C12C—H9C	0.9300	C26C—C27C	1.375 (3)
C13A—C14A	1.407 (3)	C26C—H18C	0.9300
C13B—C14B	1.414 (3)	C27A—H19A	0.9300
C13C—C14C	1.412 (3)	C27B—H19B	0.9300
C14A—C15A	1.474 (3)	C27C—H19C	0.9300
C14B—C15B	1.472 (3)	N2A—H20A	0.92 (2)
C14C—C15C	1.476 (3)	N2B—H20B	0.88 (2)
C15A—N1A	1.277 (2)	N2C—H20C	0.90 (2)
C15A—H10A	0.9300		
			
C14A—C1A—C2A	123.43 (18)	C14A—C15A—H10A	118.5
C14A—C1A—C6A	119.28 (18)	N1B—C15B—C14B	121.41 (17)
C2A—C1A—C6A	117.23 (19)	N1B—C15B—H10B	119.3
C14B—C1B—C2B	123.36 (18)	C14B—C15B—H10B	119.3
C14B—C1B—C6B	119.08 (19)	N1C—C15C—C14C	121.23 (18)
C2B—C1B—C6B	117.41 (19)	N1C—C15C—H10C	119.4
C14C—C1C—C2C	123.70 (19)	C14C—C15C—H10C	119.4
C14C—C1C—C6C	119.3 (2)	C21A—C16A—C17A	118.38 (17)
C2C—C1C—C6C	117.0 (2)	C21A—C16A—N1A	118.30 (17)
C3A—C2A—C1A	121.3 (2)	C17A—C16A—N1A	123.33 (17)
C3A—C2A—H1A	119.3	C21B—C16B—C17B	118.03 (17)
C1A—C2A—H1A	119.3	C21B—C16B—N1B	117.53 (16)
C3B—C2B—C1B	121.3 (2)	C17B—C16B—N1B	124.43 (17)
C3B—C2B—H1B	119.3	C17C—C16C—C21C	118.39 (17)
C1B—C2B—H1B	119.3	C17C—C16C—N1C	118.04 (16)
C3C—C2C—C1C	121.4 (2)	C21C—C16C—N1C	123.58 (17)
C3C—C2C—H1C	119.3	C18A—C17A—C16A	120.59 (18)
C1C—C2C—H1C	119.3	C18A—C17A—H11A	119.7
C2A—C3A—C4A	121.2 (2)	C16A—C17A—H11A	119.7
C2A—C3A—H2A	119.4	C18B—C17B—C16B	120.63 (17)
C4A—C3A—H2A	119.4	C18B—C17B—H11B	119.7
C2B—C3B—C4B	120.9 (2)	C16B—C17B—H11B	119.7
C2B—C3B—H2B	119.6	C16C—C17C—C18C	121.43 (18)
C4B—C3B—H2B	119.6	C16C—C17C—H11C	119.3
C2C—C3C—C4C	121.4 (2)	C18C—C17C—H11C	119.3
C2C—C3C—H2C	119.3	C17A—C18A—C19A	121.15 (18)
C4C—C3C—H2C	119.3	C17A—C18A—H12A	119.4
C5A—C4A—C3A	119.5 (2)	C19A—C18A—H12A	119.4
C5A—C4A—H3A	120.2	C17B—C18B—C19B	121.42 (17)
C3A—C4A—H3A	120.2	C17B—C18B—H12B	119.3
C5B—C4B—C3B	119.8 (2)	C19B—C18B—H12B	119.3
C5B—C4B—H3B	120.1	C17C—C18C—C19C	120.67 (18)
C3B—C4B—H3B	120.1	C17C—C18C—H12C	119.7
C5C—C4C—C3C	119.5 (2)	C19C—C18C—H12C	119.7
C5C—C4C—H3C	120.3	N2A—C19A—C18A	117.58 (17)
C3C—C4C—H3C	120.3	N2A—C19A—C20A	124.68 (18)
C4A—C5A—C6A	121.6 (2)	C18A—C19A—C20A	117.64 (17)
C4A—C5A—H4A	119.2	N2B—C19B—C20B	124.79 (17)
C6A—C5A—H4A	119.2	N2B—C19B—C18B	117.73 (17)
C4B—C5B—C6B	121.6 (2)	C20B—C19B—C18B	117.38 (17)
C4B—C5B—H4B	119.2	N2C—C19C—C18C	125.75 (18)
C6B—C5B—H4B	119.2	N2C—C19C—C20C	116.84 (17)
C4C—C5C—C6C	121.4 (2)	C18C—C19C—C20C	117.39 (17)
C4C—C5C—H4C	119.3	C21A—C20A—C19A	120.66 (18)
C6C—C5C—H4C	119.3	C21A—C20A—H13A	119.7
C7A—C6A—C5A	121.79 (19)	C19A—C20A—H13A	119.7
C7A—C6A—C1A	119.2 (2)	C21B—C20B—C19B	120.69 (18)
C5A—C6A—C1A	119.0 (2)	C21B—C20B—H13B	119.7
C7B—C6B—C5B	121.58 (19)	C19B—C20B—H13B	119.7
C7B—C6B—C1B	119.42 (19)	C21C—C20C—C19C	121.62 (17)
C5B—C6B—C1B	119.0 (2)	C21C—C20C—H14C	119.2
C7C—C6C—C5C	122.0 (2)	C19C—C20C—H14C	119.2
C7C—C6C—C1C	118.7 (2)	C16A—C21A—C20A	121.21 (18)
C5C—C6C—C1C	119.2 (2)	C16A—C21A—H14A	119.4
C6A—C7A—C8A	122.54 (19)	C20A—C21A—H14A	119.4
C6A—C7A—H5A	118.7	C20B—C21B—C16B	121.40 (18)
C8A—C7A—H5A	118.7	C20B—C21B—H14B	119.3
C6B—C7B—C8B	122.51 (19)	C16B—C21B—H14B	119.3
C6B—C7B—H5B	118.7	C20C—C21C—C16C	120.38 (18)
C8B—C7B—H5B	118.7	C20C—C21C—H13C	119.8
C8C—C7C—C6C	122.8 (2)	C16C—C21C—H13C	119.8
C8C—C7C—H5C	118.6	C27A—C22A—C23A	117.34 (18)
C6C—C7C—H5C	118.6	C27A—C22A—N2A	125.77 (18)
C7A—C8A—C9A	121.5 (2)	C23A—C22A—N2A	116.86 (18)
C7A—C8A—C13A	119.27 (19)	C27B—C22B—C23B	117.57 (19)
C9A—C8A—C13A	119.2 (2)	C27B—C22B—N2B	125.31 (18)
C7B—C8B—C9B	121.69 (19)	C23B—C22B—N2B	117.07 (18)
C7B—C8B—C13B	119.10 (19)	C23C—C22C—N2C	126.42 (18)
C9B—C8B—C13B	119.2 (2)	C23C—C22C—C27C	117.34 (18)
C7C—C8C—C9C	122.3 (2)	N2C—C22C—C27C	116.23 (17)
C7C—C8C—C13C	119.4 (2)	C24A—C23A—C22A	121.8 (2)
C9C—C8C—C13C	118.3 (2)	C24A—C23A—H15A	119.1
C10A—C9A—C8A	121.4 (2)	C22A—C23A—H15A	119.1
C10A—C9A—H6A	119.3	C24B—C23B—C22B	121.2 (2)
C8A—C9A—H6A	119.3	C24B—C23B—H15B	119.4
C10B—C9B—C8B	121.5 (2)	C22B—C23B—H15B	119.4
C10B—C9B—H6B	119.3	C24C—C23C—C22C	120.61 (19)
C8B—C9B—H6B	119.3	C24C—C23C—H15C	119.7
C10C—C9C—C8C	122.3 (2)	C22C—C23C—H15C	119.7
C10C—C9C—H6C	118.9	C25A—C24A—C23A	120.6 (2)
C8C—C9C—H6C	118.9	C25A—C24A—H16A	119.7
C9A—C10A—C11A	120.1 (2)	C23A—C24A—H16A	119.7
C9A—C10A—H7A	120.0	C25B—C24B—C23B	120.9 (2)
C11A—C10A—H7A	120.0	C25B—C24B—H16B	119.6
C9B—C10B—C11B	119.7 (2)	C23B—C24B—H16B	119.6
C9B—C10B—H7B	120.1	C25C—C24C—C23C	121.4 (2)
C11B—C10B—H7B	120.1	C25C—C24C—H16C	119.3
C9C—C10C—C11C	119.4 (2)	C23C—C24C—H16C	119.3
C9C—C10C—H7C	120.3	C26A—C25A—C24A	118.2 (2)
C11C—C10C—H7C	120.3	C26A—C25A—H17A	120.9
C12A—C11A—C10A	121.0 (2)	C24A—C25A—H17A	120.9
C12A—C11A—H8A	119.5	C24B—C25B—C26B	118.7 (2)
C10A—C11A—H8A	119.5	C24B—C25B—H17B	120.7
C12B—C11B—C10B	121.4 (2)	C26B—C25B—H17B	120.7
C12B—C11B—H8B	119.3	C24C—C25C—C26C	118.6 (2)
C10B—C11B—H8B	119.3	C24C—C25C—H17C	120.7
C12C—C11C—C10C	120.8 (3)	C26C—C25C—H17C	120.7
C12C—C11C—H8C	119.6	C25A—C26A—C27A	121.8 (2)
C10C—C11C—H8C	119.6	C25A—C26A—H18A	119.1
C11A—C12A—C13A	120.9 (2)	C27A—C26A—H18A	119.1
C11A—C12A—H9A	119.5	C25B—C26B—C27B	121.2 (2)
C13A—C12A—H9A	119.5	C25B—C26B—H18B	119.4
C11B—C12B—C13B	121.0 (2)	C27B—C26B—H18B	119.4
C11B—C12B—H9B	119.5	C27C—C26C—C25C	120.5 (2)
C13B—C12B—H9B	119.5	C27C—C26C—H18C	119.7
C11C—C12C—C13C	121.4 (2)	C25C—C26C—H18C	119.7
C11C—C12C—H9C	119.3	C22A—C27A—C26A	120.2 (2)
C13C—C12C—H9C	119.3	C22A—C27A—H19A	119.9
C14A—C13A—C12A	123.68 (19)	C26A—C27A—H19A	119.9
C14A—C13A—C8A	118.98 (19)	C26B—C27B—C22B	120.4 (2)
C12A—C13A—C8A	117.34 (19)	C26B—C27B—H19B	119.8
C14B—C13B—C12B	123.45 (18)	C22B—C27B—H19B	119.8
C14B—C13B—C8B	119.23 (19)	C26C—C27C—C22C	121.4 (2)
C12B—C13B—C8B	117.29 (18)	C26C—C27C—H19C	119.3
C14C—C13C—C12C	123.42 (19)	C22C—C27C—H19C	119.3
C14C—C13C—C8C	118.7 (2)	C15A—N1A—C16A	117.97 (16)
C12C—C13C—C8C	117.7 (2)	C15B—N1B—C16B	119.33 (16)
C13A—C14A—C1A	120.68 (18)	C15C—N1C—C16C	119.17 (16)
C13A—C14A—C15A	121.49 (18)	C19A—N2A—C22A	130.68 (17)
C1A—C14A—C15A	117.74 (17)	C19A—N2A—H20A	114.2 (14)
C13B—C14B—C1B	120.57 (18)	C22A—N2A—H20A	114.1 (13)
C13B—C14B—C15B	121.34 (18)	C19B—N2B—C22B	130.67 (17)
C1B—C14B—C15B	117.97 (18)	C19B—N2B—H20B	114.1 (14)
C1C—C14C—C13C	121.10 (19)	C22B—N2B—H20B	114.5 (14)
C1C—C14C—C15C	120.96 (19)	C19C—N2C—C22C	132.68 (17)
C13C—C14C—C15C	117.76 (19)	C19C—N2C—H20C	112.0 (12)
N1A—C15A—C14A	122.92 (18)	C22C—N2C—H20C	115.3 (12)
N1A—C15A—H10A	118.5		
			
C14A—C1A—C2A—C3A	179.77 (19)	C2B—C1B—C14B—C15B	2.5 (3)
C6A—C1A—C2A—C3A	−3.1 (3)	C6B—C1B—C14B—C15B	−172.84 (17)
C14B—C1B—C2B—C3B	−176.94 (19)	C2C—C1C—C14C—C13C	−177.79 (18)
C6B—C1B—C2B—C3B	−1.5 (3)	C6C—C1C—C14C—C13C	0.9 (3)
C14C—C1C—C2C—C3C	179.74 (19)	C2C—C1C—C14C—C15C	7.2 (3)
C6C—C1C—C2C—C3C	1.0 (3)	C6C—C1C—C14C—C15C	−174.09 (18)
C1A—C2A—C3A—C4A	0.8 (3)	C12C—C13C—C14C—C1C	−176.86 (19)
C1B—C2B—C3B—C4B	1.0 (3)	C8C—C13C—C14C—C1C	−0.5 (3)
C1C—C2C—C3C—C4C	−0.8 (3)	C12C—C13C—C14C—C15C	−1.7 (3)
C2A—C3A—C4A—C5A	1.6 (3)	C8C—C13C—C14C—C15C	174.68 (17)
C2B—C3B—C4B—C5B	0.0 (3)	C13A—C14A—C15A—N1A	−41.9 (3)
C2C—C3C—C4C—C5C	0.1 (3)	C1A—C14A—C15A—N1A	134.6 (2)
C3A—C4A—C5A—C6A	−1.4 (3)	C13B—C14B—C15B—N1B	−44.8 (3)
C3B—C4B—C5B—C6B	−0.4 (3)	C1B—C14B—C15B—N1B	131.35 (19)
C3C—C4C—C5C—C6C	0.4 (3)	C1C—C14C—C15C—N1C	48.9 (3)
C4A—C5A—C6A—C7A	177.4 (2)	C13C—C14C—C15C—N1C	−126.3 (2)
C4A—C5A—C6A—C1A	−0.9 (3)	C21A—C16A—C17A—C18A	−4.9 (3)
C14A—C1A—C6A—C7A	2.0 (3)	N1A—C16A—C17A—C18A	175.18 (17)
C2A—C1A—C6A—C7A	−175.28 (18)	C21B—C16B—C17B—C18B	−5.6 (3)
C14A—C1A—C6A—C5A	−179.59 (17)	N1B—C16B—C17B—C18B	173.92 (17)
C2A—C1A—C6A—C5A	3.1 (3)	C21C—C16C—C17C—C18C	−4.0 (3)
C4B—C5B—C6B—C7B	178.4 (2)	N1C—C16C—C17C—C18C	175.62 (17)
C4B—C5B—C6B—C1B	−0.1 (3)	C16A—C17A—C18A—C19A	−0.3 (3)
C14B—C1B—C6B—C7B	−1.9 (3)	C16B—C17B—C18B—C19B	0.4 (3)
C2B—C1B—C6B—C7B	−177.54 (18)	C16C—C17C—C18C—C19C	3.3 (3)
C14B—C1B—C6B—C5B	176.68 (18)	C17A—C18A—C19A—N2A	−178.59 (17)
C2B—C1B—C6B—C5B	1.0 (3)	C17A—C18A—C19A—C20A	4.9 (3)
C4C—C5C—C6C—C7C	−179.1 (2)	C17B—C18B—C19B—N2B	−178.26 (17)
C4C—C5C—C6C—C1C	−0.2 (3)	C17B—C18B—C19B—C20B	5.1 (3)
C14C—C1C—C6C—C7C	−0.4 (3)	C17C—C18C—C19C—N2C	−178.85 (18)
C2C—C1C—C6C—C7C	178.43 (18)	C17C—C18C—C19C—C20C	−0.4 (3)
C14C—C1C—C6C—C5C	−179.29 (18)	N2A—C19A—C20A—C21A	179.34 (18)
C2C—C1C—C6C—C5C	−0.5 (3)	C18A—C19A—C20A—C21A	−4.4 (3)
C5A—C6A—C7A—C8A	179.69 (19)	N2B—C19B—C20B—C21B	178.23 (19)
C1A—C6A—C7A—C8A	−2.0 (3)	C18B—C19B—C20B—C21B	−5.4 (3)
C5B—C6B—C7B—C8B	−178.80 (19)	N2C—C19C—C20C—C21C	176.77 (17)
C1B—C6B—C7B—C8B	−0.3 (3)	C18C—C19C—C20C—C21C	−1.9 (3)
C5C—C6C—C7C—C8C	178.2 (2)	C17A—C16A—C21A—C20A	5.3 (3)
C1C—C6C—C7C—C8C	−0.6 (3)	N1A—C16A—C21A—C20A	−174.70 (18)
C6A—C7A—C8A—C9A	−179.57 (19)	C19A—C20A—C21A—C16A	−0.7 (3)
C6A—C7A—C8A—C13A	−0.1 (3)	C19B—C20B—C21B—C16B	0.3 (3)
C6B—C7B—C8B—C9B	−177.34 (19)	C17B—C16B—C21B—C20B	5.3 (3)
C6B—C7B—C8B—C13B	0.9 (3)	N1B—C16B—C21B—C20B	−174.25 (18)
C6C—C7C—C8C—C9C	178.9 (2)	C19C—C20C—C21C—C16C	1.2 (3)
C6C—C7C—C8C—C13C	1.1 (3)	C17C—C16C—C21C—C20C	1.7 (3)
C7A—C8A—C9A—C10A	177.7 (2)	N1C—C16C—C21C—C20C	−177.85 (17)
C13A—C8A—C9A—C10A	−1.7 (3)	C27A—C22A—C23A—C24A	0.2 (3)
C7B—C8B—C9B—C10B	177.1 (2)	N2A—C22A—C23A—C24A	178.4 (2)
C13B—C8B—C9B—C10B	−1.2 (3)	C27B—C22B—C23B—C24B	1.2 (3)
C7C—C8C—C9C—C10C	−177.4 (2)	N2B—C22B—C23B—C24B	178.7 (2)
C13C—C8C—C9C—C10C	0.5 (3)	N2C—C22C—C23C—C24C	−178.25 (19)
C8A—C9A—C10A—C11A	−0.3 (3)	C27C—C22C—C23C—C24C	0.3 (3)
C8B—C9B—C10B—C11B	0.3 (3)	C22A—C23A—C24A—C25A	1.1 (4)
C8C—C9C—C10C—C11C	0.9 (4)	C22B—C23B—C24B—C25B	0.3 (4)
C9A—C10A—C11A—C12A	1.4 (3)	C22C—C23C—C24C—C25C	1.5 (3)
C9B—C10B—C11B—C12B	0.8 (3)	C23A—C24A—C25A—C26A	−1.1 (3)
C9C—C10C—C11C—C12C	−0.9 (4)	C23B—C24B—C25B—C26B	−1.1 (4)
C10A—C11A—C12A—C13A	−0.4 (3)	C23C—C24C—C25C—C26C	−1.8 (3)
C10B—C11B—C12B—C13B	−1.0 (3)	C24A—C25A—C26A—C27A	−0.3 (3)
C10C—C11C—C12C—C13C	−0.6 (3)	C24B—C25B—C26B—C27B	0.6 (3)
C11A—C12A—C13A—C14A	179.49 (19)	C24C—C25C—C26C—C27C	0.3 (3)
C11A—C12A—C13A—C8A	−1.5 (3)	C23A—C22A—C27A—C26A	−1.6 (3)
C7A—C8A—C13A—C14A	2.2 (3)	N2A—C22A—C27A—C26A	−179.6 (2)
C9A—C8A—C13A—C14A	−178.39 (18)	C25A—C26A—C27A—C22A	1.7 (3)
C7A—C8A—C13A—C12A	−176.89 (18)	C25B—C26B—C27B—C22B	0.9 (3)
C9A—C8A—C13A—C12A	2.6 (3)	C23B—C22B—C27B—C26B	−1.7 (3)
C11B—C12B—C13B—C14B	−177.72 (19)	N2B—C22B—C27B—C26B	−179.06 (19)
C11B—C12B—C13B—C8B	0.1 (3)	C25C—C26C—C27C—C22C	1.4 (3)
C7B—C8B—C13B—C14B	0.6 (3)	C23C—C22C—C27C—C26C	−1.7 (3)
C9B—C8B—C13B—C14B	178.89 (17)	N2C—C22C—C27C—C26C	176.96 (19)
C7B—C8B—C13B—C12B	−177.36 (17)	C14A—C15A—N1A—C16A	−178.47 (17)
C9B—C8B—C13B—C12B	1.0 (3)	C21A—C16A—N1A—C15A	141.24 (19)
C11C—C12C—C13C—C14C	178.3 (2)	C17A—C16A—N1A—C15A	−38.8 (3)
C11C—C12C—C13C—C8C	2.0 (3)	C14B—C15B—N1B—C16B	−176.35 (17)
C7C—C8C—C13C—C14C	−0.5 (3)	C21B—C16B—N1B—C15B	147.60 (18)
C9C—C8C—C13C—C14C	−178.42 (19)	C17B—C16B—N1B—C15B	−31.9 (3)
C7C—C8C—C13C—C12C	176.07 (19)	C14C—C15C—N1C—C16C	178.31 (17)
C9C—C8C—C13C—C12C	−1.9 (3)	C17C—C16C—N1C—C15C	−137.29 (19)
C12A—C13A—C14A—C1A	176.92 (18)	C21C—C16C—N1C—C15C	42.3 (3)
C8A—C13A—C14A—C1A	−2.1 (3)	C18A—C19A—N2A—C22A	165.76 (19)
C12A—C13A—C14A—C15A	−6.8 (3)	C20A—C19A—N2A—C22A	−18.0 (3)
C8A—C13A—C14A—C15A	174.26 (17)	C27A—C22A—N2A—C19A	−12.1 (3)
C2A—C1A—C14A—C13A	177.12 (18)	C23A—C22A—N2A—C19A	169.9 (2)
C6A—C1A—C14A—C13A	0.0 (3)	C20B—C19B—N2B—C22B	−16.8 (3)
C2A—C1A—C14A—C15A	0.7 (3)	C18B—C19B—N2B—C22B	166.81 (19)
C6A—C1A—C14A—C15A	−176.46 (17)	C27B—C22B—N2B—C19B	−11.2 (3)
C12B—C13B—C14B—C1B	175.06 (18)	C23B—C22B—N2B—C19B	171.4 (2)
C8B—C13B—C14B—C1B	−2.7 (3)	C18C—C19C—N2C—C22C	−6.2 (3)
C12B—C13B—C14B—C15B	−8.9 (3)	C20C—C19C—N2C—C22C	175.27 (18)
C8B—C13B—C14B—C15B	173.37 (17)	C23C—C22C—N2C—C19C	−12.3 (3)
C2B—C1B—C14B—C13B	178.77 (18)	C27C—C22C—N2C—C19C	169.19 (19)
C6B—C1B—C14B—C13B	3.4 (3)		

### Hydrogen-bond geometry (Å, º)

*Cg*2, *Cg*4, *Cg*10,*Cg*11, *Cg*12, *Cg*13,*Cg*18, *Cg*20 and *Cg*21are the centroids of the C1*A*–C14*A*, C16*A*–C21*A*, C1*B*–C14*B*, C8*B*–C13*B*, C16*B*–C21*B*, C22*B*–C27*B*, C1*C*–C14*C*, C22*C*–C27*C* and C1*C*–C8*C*rings, respectively.

**Table:**

*D*—H···*A*	*D*—H	H···*A*	*D*···*A*	*D*—H···*A*
C12*A*—H9*A*···N1*A*	0.93	2.44	2.989 (3)	118
C12*B*—H9*B*···N1*B*	0.93	2.47	3.006 (3)	117
C2*C*—H1*C*···N1*C*	0.93	2.51	3.032 (3)	116
N2*A*—H20*A*···N1*A*^i^	0.92 (2)	2.28 (2)	3.147 (2)	158.4 (18)
N2*B*—H20*B*···N1*C*^ii^	0.88 (2)	2.19 (2)	3.056 (2)	166.2 (19)
N2*C*—H20*C*···N1*B*	0.90 (2)	2.22 (2)	3.094 (2)	163.2 (17)
C2*A*—H1*A*···*Cg*13^iii^	0.93	2.93	3.7248 (3)	144
C2*B*—H1*B*···*Cg*21^iv^	0.93	2.91	3.6227 (3)	134
C4*A*—H3*A*···*Cg*21^v^	0.93	2.75	3.6728 (3)	175
C10*A*—H7*A*···*Cg*12^ii^	0.93	2.80	3.5075 (3)	134
C17*A*—H11*A*···*Cg*18^ii^	0.93	2.65	3.5119 (3)	154
C17*B*—H11*B*···*Cg*10^v^	0.93	2.79	3.6243 (3)	150
C17*B*—H11*B*···*Cg*11^v^	0.93	2.89	3.7248 (3)	150
C21*C*—H13*C*···*Cg*2^vi^	0.93	2.86	3.7513 (3)	162
C23*A*—H15*A*···*Cg*2^i^	0.93	2.85	3.7253 (3)	157
C23*B*—H15*B*···*Cg*18^ii^	0.93	2.87	3.7057 (3)	149
C25*A*—H17*A*···*Cg*20^vii^	0.93	2.96	3.6262 (3)	130
C25*B*—H17*B*···*Cg*4^viii^	0.93	3.00	3.6476 (3)	128
C25*C*—H17*C*···*Cg*12^iv^	0.93	2.84	3.6263 (3)	143

Symmetry codes: (i) *x*, −*y*+3/2, *z*−1/2; (ii) *x*, *y*, *z*+1; (iii) *x*−1, *y*, *z*−1; (iv) −*x*+2, −*y*+1, −*z*+1; (v) −*x*+1, −*y*+1, −*z*+1; (vi) *x*, *y*, *z*−1; (vii) *x*−1, −*y*+3/2, *z*+1/2; (viii) *x*+1, *y*, *z*.
